# Demorest’s Monthly

**Published:** 1874-03

**Authors:** 


DEMOREST'S MONTHLY.—This mirror of fashion and elegant manners comes to our sanctum, in its spring dress, a "thing of beauty." Doctor's wives, like all other men's wives, cannot well afford to get along without Demorest. They ought to have it. Price, $3.00. Address W. Jennings Demorest, 838 Broadway, N. Y.

